# Effect of laser welding on microstructure and mechanical behaviour of dual phase 600 steel sheets

**DOI:** 10.1016/j.heliyon.2021.e08601

**Published:** 2021-12-15

**Authors:** Vinicius Machado Mansur, Raquel Alvim de Figueiredo Mansur, Sheila Medeiros de Carvalho, Rafael Humberto Mota de Siqueira, Milton Sergio Fernandes de Lima

**Affiliations:** a Photonics Divison, Institute for Advanced Studies, 12228-001, Sao Jose dos Campos, SP, Brazil; b Department of Mechanical Engineering, Federal University of Espirito Santo, 29075-910, Vitoria, ES, Brazil

**Keywords:** Laser beam welding, Dual-phase steels, Formability

## Abstract

Dual Phase steels are one of the most used advanced high-strength materials in the industry, due to its combination of a ductile ferritic matrix and disperse hard martensite islands, which provide outstanding mechanical properties for components to be cold stamped. This work investigated fiber laser welding applicability in Dual Phase 600 1.6 mm thick steel sheets, evaluating potential welding impacts on properties of the material for industrial applications. A first set of bead-on-plate welds was generated to define best parameters for subsequent tests. A second set was prepared, consisting of butt joints welded in the optimized condition. Weld microstructure was characterized as 100% martensitic at fusion zone (FZ), with growing fractions of ferrite at Heat Affected Zone (HAZ) as one moves away from fusion line. Hardness is around 60% higher at FZ than at BM, being maximum at supercritical HAZ due to its highly refined microstructure and HAZ softening was not observed. Tensile and Erichsen cupping tests presented similar strength results between welded and non-welded specimens, with slight ductility reduction. Finally, numeric simulations based on Finite Element Analysis were carried out to estimate temperature evolution, phase proportions, residual stresses and distortion levels, achieving excellent agreement with experimental results.

## Highlights

• The laser welds are defect-free and presented a major fraction of martensite microconstituent in both fusion and heat-affected zone.
• In contrast to what is reported in the literature, the heat-affected zone did not show a decrease in hardness due to the tempering.
• The results of the uniaxial tensile tests and Erichsen drawability show that welded sheets are applicable for cold stamping.

## 1 Introduction

Steel has being used in almost all economic segments, as aerospace (Garrison, 1990; Sakai et al., 2015), automotive (Bouaziz et al., 2013; Galán et al., 2012; Kuziak et al., 2008) and oil and gas industries (Chail and Kangas, 2016; Sharma and Maheshwari, 2017), due to its remarkable combination between mechanical properties, low cost and ease of transformation. Besides that, steels are considered the most recycled materials in the world (Keeler et al., 2017), which can mitigate ambiental impacts of the production cycle as a whole. On the last decades, growing emphasis has been given to the so called Advanced High Strength Steels (AHSS), especially in the automotive industry. Due to their higher tensile strength and work hardening rates, those steels allow the fabrication of parts that are stronger and lighter at the same time, which translates into increased performance, fuel economy, safety improvements and other advantages (Keeler et al., 2017; Kuziak et al., 2008; Matlock and Speer, 2009).

One of the most used AHSS in the automotive industry is the Dual Phase steel, which basically consists of a ferritic matrix with martensite islands, in variable proportions depending on steel grade being used (Alves et al., 2018; Correard et al., 2016; Farabi et al., 2010). The combination of ferrite ductility and martensite strength gives Dual Phase steels excellent mechanical properties for parts to be processed by forming, as elevated Ultimate Tensile Strength (UTS) and work hardening coefficient, as well as low yield strength/tensile strength ratio (Dong et al., 2014).

The use of dual phase steel sheets in industrial applications invariably involves joining processes, whether before or after its conformation (Keeler et al., 2017). Among these processes, one of the most common is laser welding that, due to its intrinsic characteristics, provides excellent mechanical continuity in the welded joint, in addition to being easily automated and having great application flexibility (Dong et al., 2014). However, high temperatures achieved during welding cause transformations in material microstructure (Alves et al., 2018) that can reduce component life or even lead to catastrophic failures. In this way, it is of fundamental importance to use optimized welding procedures for each specific condition, based on research results and controlled experiments.

### 1.1 Laser welding of dual phase sheets

The application of laser welding on DP steels leads to fusion of material in the central region of the welded joint. This region, which peak temperature during welding exceeds the fusion point of the material, constitutes the Fusion Zone (FZ). As this region experiences intense heating and cooling thermal cycles, its microstructure is almost entirely martensitic, with hardness levels in the order of 1.5–2 times the hardness of base metal (Dong et al., 2014; Farabi et al., 2010; Xia et al., 2008). Some authors observed, in addition to martensite, portions of ferrite and bainite in this region (Alves et al., 2018; Correard et al., 2016; Zhao et al., 2013). In general, FZ hardness presents directly linear relationship with the carbon equivalent of the steel (Zhao et al., 2013).

Near FZ, on both sides of the joint, where no fusion is observed, solid-state phase transformations take place due to welding heat input, constituting the so-called Heat Affected Zone (HAZ). Within HAZ, the region that is closest to FZ is called “supercritical” as it is fully austenitized during welding. Due to the subsequent fast cooling, austenite completely transforms to martensite after welding. Farther away from the weld, the next region is called “intercritical” because its lower temperatures (between Ac1 and Ac3 lines of Fe–C phase diagram) lead to partial austenitization of the material, later originating a mixed microstructure of areas not dissolved from the base metal (ferrite and martensite) associated with some martensite generated by welding. In summary, HAZ intercritical and supercritical regions contain fine martensite and undissolved ferrite whose volumetric fraction increases with distance from FZ (Dong et al., 2014). Depending on welding parameters and characterization methodology, small amounts of bainite can also be observed (Farabi et al., 2010).

The HAZ region that is closest to BM is called "subcritical" because there is no austenitization. However, tempering of the pre-existing martensite in the base metal can occur, which can lead, depending on the welding parameters and the type of DP steel, to hardness levels below those obtained on base metal (Dong et al., 2014; Farabi et al., 2010; Zhao et al., 2013).

A study (Alves et al., 2018) using laser welding of a DP1000 steel indicated, in addition to the presence of martensite, ferrite and bainite in the welded joint, small amounts of retained austenite. An increasing volume fraction of this constituent was observed from the FZ towards the HAZ, reaching a maximum of 2.9% between intercritical and subcritical HAZ. The author concluded that the presence of retained austenite, along with martensite tempering, contributed to the softening effect.

The ratio between laser power and welding speed is, for practical purposes, what is called heat input. This approach considers a moving heat source along plate surface, without any losses at external surfaces by convection or thermal radiation (Kouadri-Henni, 2017). Considering a constant laser beam diameter at part surface, heat input increases with increasing laser power and decreasing welding speed (Bandyopadhyay et al., 2016). Heat input not only affects softening phenomenon, but every aspect of the welding. Consequently, process parameters shall be carefully adjusted for obtaining an adequate weld.

A heat input that is too low, due to excessive welding speed or insufficient power, may cause a lack of penetration in the welded joint (Fernandes et al., 2017), along with pores at FZ due to keyhole collapse and solidification processes at weld pool that restrict the flow of molten metal (Alves et al., 2018). An extremely high heat input, on the other hand, generates larger weld beads (Wang et al., 2017), thus promoting HAZ softening and leading to a less refined microstructure. Both effects are detrimental for the mechanical properties of welded joint (Xie et al., 2017).

Previous research work pointed out a correlation between welding speed, laser power, and FZ and HAZ dimensions. A lower speed favours weld penetration, but also increase heat dissipation in the transverse direction to the weld, which turns into wider welds. Considering a constant welding speed, increasing the power has the same effect of increasing weld width and penetration (Alves et al., 2018).

As mentioned before, hardness in the weld tends to be higher than in the base metal due to the higher fraction of hard microconstituents, such as bainite and martensite. Thus, if welding procedure does not induce softening in the HAZ, the welded joint will present similar or even higher mechanical resistance in comparison to the base metal. This increase in strength can be verified through uniaxial tensile tests, which invariably will lead welded samples to rupture far from the weld, obtaining similar results to those of unwelded specimens in terms of yield and tensile strength. Uniform elongation values tend to be reduced, because the weld bead has high hardness and is therefore less ductile. However, since the weld width is usually small when compared to total size of the component, this reduction is not significant enough to impair its formability (Evin and Tomáš, 2017; Fernandes et al., 2017; Xie et al., 2017).

However, mechanical properties can be actually harmed if the laser welding conditions lead to the occurrence of the softening phenomenon at subcritical HAZ. In this case, a weakened region is formed near the welded joint. This condition can be evidenced through uniaxial tensile tests, where the specimens start to break preferentially in the softened region, with very low elongation and tensile strength levels below those obtained in samples without welding, sometimes even below minimum tensile strength specified for that material (Gao et al., 2018; Wang et al., 2016).

Xu et al. (2013) employed a laser source with high energy density (power of 6 kW and beam diameter of 0.6 mm) on DP980 steel samples, obtaining welds with greatly reduced HAZ and FZ (200–300 μm and 400–500 μm, respectively). Although the phenomenon of softening at HAZ was observed, and all samples consistently failed in this region, the mechanical strength of the joint remained practically unchanged, since the tensile tests on welded and non-welded samples obtained very similar yield and tensile strength values. This fact can be attributed to the minor width of the softened region. Elongation on welded samples, however, was at least 60% lower than that of base metal because, once the yield point is reached at the lower hardness zone; the subsequent plastic deformation is concentrated there, leading to necking and premature failure with reduced total elongation.

Farabi et al. (2010) conducted tensile tests on samples welded with a diode laser (12 mm × 0.9 mm rectangular beam, 239 J/mm heat input). In addition to the fact that all welded samples ruptured at HAZ softened region, a discontinuous yield strength, which is not usual on DP steel plates as mentioned before, was observed. This change in yield behaviour can be attributed to the diffusion of interstitial atoms such as carbon and nitrogen, promoted by the high heat input, to positions of high energy at the core of edge dislocations, creating a pinning effect that restrains dislocation movement to start the plastic deformation. Once the dislocations overcome this potential barrier, their slip starts to occur at a lower stress, causing a yield discontinuity. The study also observed uniform elongation values at least 50% below those obtained on non-welded samples, which were attributed to localized deformation at HAZ softened region. The existence of a predominantly martensitic weld beads, with FZ around 4 mm wide, may also have contributed to elongation reduction.

Fernandes et al. (2017) performed pulsed Nd:YAG laser welds on DP600 steel plates with a thickness of 0.8 mm. Tensile tests were performed on five samples, welded under the best conditions determined by the study. Optical correlation during the test was performed by a software called Aramis to map specimen deformation in three dimensions. The results showed lower strain levels in the weld bead, which can be attributed to the hardness almost twice as high as in the base metal. As a result, all samples failed far from the welded joint, except for sample 4, which broke in HAZ region softened by martensite tempering.

Material modifications caused by laser welding can also influence the formability of welded plates. Studies conducted by Bandyopadhyay et al. (2017) on laser welded DP600 steel sheets showed that the increase in FZ hardness due to martensitic transformations reduced the formability of the material about 28%, when compared to the unwelded specimen.

Li et al. (2013) evaluated the effects of weld bead position and geometry (curved or linear) on the formability of High Strength Low Alloy (HSLA), DP600 and DP980 steels, using a diode laser beam with rectangular shape (12 mm x 0, 9 mm). The phenomenon of softening at subcritical HAZ was observed and exhibited a strong correlation with the fracture site during the Limited Dome Height (LDH) tests, since the failure occurred consistently in this region. Failure occurred in the internal region of curved welds, where more intense heat input led to an average softening of 11 HV for DP 600 and 132 HV for DP 980.

Experiments conducted by Wang et al. (2017) on DP780 sheets with a thickness of 1.5 mm showed that, in the welding heat input range between 18 and 42 J/mm, the fracture during the Erichsen formability test occurred perpendicular to the weld, with an Erichsen index (IE) around 95% of that obtained at base metal. At the sample welded at 66 J/mm, however, the fracture propagated along a 600 μm wide softened region, and the formability was around 80% of that observed on non-welded samples. A critical size of the softened zone (SZ) was estimated at 540 μm, since no relevant damage on the weld joint mechanical properties was observed on SZ below that size. This information provides some guidance for designing welding parameters and pre-evaluate the welded joint quality.

Mitra et al. (2020a) performed laser welding on DP600 samples containing notches inserted at weld HAZ, to study the fracture behaviour in this region by means of tensile tests. Results evidenced a direct relation between welding speed and increase in the size of the cavities at fracture surface. At the highest tested velocity, a transition from ductile to a mixed (ductile/brittle) mode of fracture was observed. At fusion zone, the increase in welding speed significantly reduced the equiaxiality of austenitic grains, which, according to the authors, could be detrimental to component formability.

During laser welding, a narrow region of material is suddenly heated, melted and resolidified locally. After subsequent cooling, the union of the parts is achieved. The elevated imposed thermal gradients strongly affect the mechanical properties of the material by promoting heterogeneous volumetric expansions and contractions that cause residual stresses. Once they reach a critical limit, these stresses are relieved by permanent deformation until a structurally balanced stress state is achieved, causing undesirable distortion effects on welded components (Deshpande et al., 2011; Tsirkas et al., 2003).

Kouadri-Henni et al. (2017) studied the distribution of residual stresses in a DP600 laser welded steel, comparing experimental values, obtained by neutron diffraction technique, and finite element simulations. Two models were used in the simulation: elastoplastic and visco-elastoplastic, the latter considering the effect of volumetric expansion induced by phase transformations. The results indicated a high level of residual tensile stresses at weld FZ, in good convergence between experimental results and numerical simulations. The study concluded that the main factor of influence on residual stresses is the temperature evolution during welding.

Laser welding of thin DP steel plates induces more intense residual stresses in the longitudinal direction (weld axis), with tensile stresses close to material yield strength at the center of the weld, which progressively decrease and become slightly compressive at base material. In the transverse direction, residual stresses present a similar pattern, tensile at the center and compressive at the edges of the weld, although at lower levels when compared to the longitudinal ones (up to 8 times lower) (Carrizalez-Vazquez and Pérez-Medina, 2019).

Studies conducted by Derakhshan et al. (2018) indicated a tendency to increase the tensile stresses in the weld with increasing cooling rates, by using welding techniques with lower heat input. This relationship was attributed to the fact that higher cooling rates lead to an increase in the hardness of the weld bead and, consequently, its resistance to restriction effect applied by adjacent regions during solidification and phase transformations, resulting in higher residual stress levels. The amount of distortion, on the other hand, presented an inverse trend, as the samples welded at the lowest heat input obtained the lowest level of distortion, due to the narrower HAZ. For all samples, the maximum deflection was observed at the center of the weld, which was the region subjected to the most intense heat input.

Weld tests performed by Yan et al. (2019) in DP780 steel samples obtained similar results, showing a reduction in component distortion with the use of higher welding speeds and lower welding currents, being the former parameter considered more influential than the latter.

The same directly proportional relationship between the level of residual stresses and the cooling rate was observed in the tests conducted by Mitra et al. (2020b). In this work, a set of 1.2 mm thick DP600 steel sheets was welded with fiber laser equipment, using eight different welding speeds while changing power to maintain heat input around 25 J/mm. The results showed that an increase in welding speed generates higher residual stresses, above material yield strength at the highest speeds, as well as less dispersion on measurements, which were performed by X-ray diffraction. The explanation proposed by the authors lies in the fact that an increase in welding speed changes gradually liquid metal pool shape from elliptical to teardrop. As the principal heat flow direction is normal to solid-liquid interface, a teardrop-like liquid pool tends to transfer most of its heat to the cooler base metal, providing comparatively higher and less variable cooling rates.

Laser welding processes are characterized by high concentrated beam energy, which provides welds with very small dimensions. Thus, it is difficult to make accurate measurements of temperature evolution at FZ and HAZ during welding. It is a relatively complex process as it involves thermal, mechanical and metallurgical phenomena, such as phase transformations and heat flux. In addition, the mechanical and thermal material properties also vary with temperature (Sankaranarayanasamy et al., 2008).

In order to try to understand the relationship between all these variables and assist the development of welding procedures, several authors have developed computer simulations through finite element analysis (FEA), which are used to estimate residual stress, distortion and plastic deformation in welded joints. These simulations can partially replace experimental sample tests, lowering project costs. By optimizing the welding parameters, they provide better quality to the welded products, avoiding misalignment and unexpected failures during operation (Carrizalez-Vazquez and Pérez-Medina, 2019). Some works have pointed out as the most important aspects of laser welding simulation the introduction of metallurgical transformations in the model and the correct keyhole representation (Tsirkas et al., 2003).

One of the most used tools for laser welding simulation is the SYSWELD finite element package. This software, property of ESI group, simulates welding thermal phenomena welding through the transient model of heat conduction (Lima et al., 2017; Sankaranarayanasamy et al., 2008).

In this model, temperature profile at the weld joint is calculated at each temporal increment, which can be automatically adjusted as a function of mesh density or manually set by the user. Usually, though, time interval is a function of the previously defined temperature resolution. The simulated heat source moves along a line that determines the weld trajectory. The parameters used to describe the heat source model are the same provided on welding procedures, such as current, voltage, welding speed, thermal efficiency, etc (Kik, 2020a). If needed, these parameters are replaced by those specifically related to laser welding.

Three predefined types of heat source are normally used: Gaussian surface, double ellipsoid or conical. These models can be modified to better adapt to the expected results, being of paramount importance in this case to compare the simulations with experimental results, obtained in real welded specimens, in order to calibrate and validate developed models (Kik, 2020b). For instance, the energy transfer efficiency between the laser beam and the keyhole, called optical absorptivity, varies from zero (white body) to one (black body). Each welding condition of the same material, with the same laser beam, under different experimental conditions, leads to different absorptivities.

The modelled finite element mesh is defined as a function of the compromise between computational time and model accuracy, so it is usual to use more refined elements on the weld region and a coarser mesh in the rest of the structure (Komerla et al., 2020; Tsirkas et al., 2003).

Correard et al. (2016) simulated, through finite element analyses, dissimilar laser welding between DP600 and TRIP750 steel sheets, employing a Gaussian energy distribution, 1.5 mm keyhole penetration and 85% absorptivity. Material parameters used in the simulation were obtained from the SYSWELD software library. The simulation results allowed evaluation of microstructural profile and hardness evolution along the joint, in relatively good agreement with the actual weld.

Some authors (Carrizalez-Vazquez and Pérez-Medina, 2019) used a thermometallurgical and mechanical model to simulate laser welding on DP600 steel sheets. The simulations indicated quite consistent results between estimated thermal profile and macrographic tests on welded specimens. In addition, simulations with different welding sequences were carried out, keeping a constant heat input. The first condition consisted of carrying out the welding in a single 200 mm long pass. In the second condition, two 100 mm long passes were used to cover the entire length of the plate, waiting total cooling of the first bead before applying the next one. This second simulation indicated 5–8% residual stress reduction and 14% reduction in the level of distortions.

The purpose of this work is to investigate the applicability of laser welding in Dual Phase 600 steel sheets, evaluating potential welding effects, whether positive or negative, in the material physical and mechanical properties, acting as a subsidy for more productive, secure and trustworthy industrial practices. The coupons were made, employing different welding conditions, for the choice of optimized parameters that would promote the best results in terms of quality of the welded joint. These welded coupons were submitted to metallographic and mechanical analyses in order to evaluate their properties. As an original contribution to the state of the art, it is studied the effect of laser beam welding on the cold formability of DP600 sheets under controlled Erichsen tests and its correlation to the estimated mechanical behavior by finite elements analyses.

## 2 Materials and methodology

### 2.1 Materials

The Dual Phase (DP) grade 600 steel sheets used in this study were supplied by company *Usiminas* in as-rolled and annealed conditions, with a thickness of 1.6 mm and chemical composition as shown in Table 1 (Usiminas DP600GA standard). The mechanical properties of the material as supplied can be found in Table 2. Those data were obtained from the certificates, issued by the manufacturer for the specific batch from which samples were taken.

**Table 1:** Chemical composition of DP600 samples, % weight, Fe as balance.

**Al**	**N**	**P**	**C**	**S**	**Si**	**Mn**	**Mg**
0.032	0.0052	0.016	0.076	0.0048	0.017	1.67	<0.005
**Cu**	**Ni**	**Cr**	**Mo**	**Nb**	**Ti**	**W**	**V**
0.015	0.017	0.023	0.16	0.010	<0.005	<0.005	<0.005

**Table 2:** Mechanical properties of DP600 supplied samples.

Yield Strength (YS) (MPa)	Ultimate Tensile Strength (UTS) (MPa)	Uniform elongation (ε_u_) (%)	Maximum elongation (ε_m_) (%)
350 ± 4	635 ± 4	19.5 ± 0.5	29.0 ± 0.8

The DP 600 sheets were double hot-dip galvanized at the mill (GA, galvanneling). Coating chemical composition was characterized by atomic absorption spectrometry (AAS) by material supplier *Usiminas*. The layer mass was estimated by the initial sample weighing, followed by zinc layer removal in HCℓ solution with hexatylenetetramine corrosion inhibitor (3.5 g/ℓ) and subsequent weighing after removal. The GA coating layer is made of a Zn–Fe alloy with thickness of approximately 5 μm (35.7 g/m^2^).

### 2.2 Welding procedure

**Figure 1:**
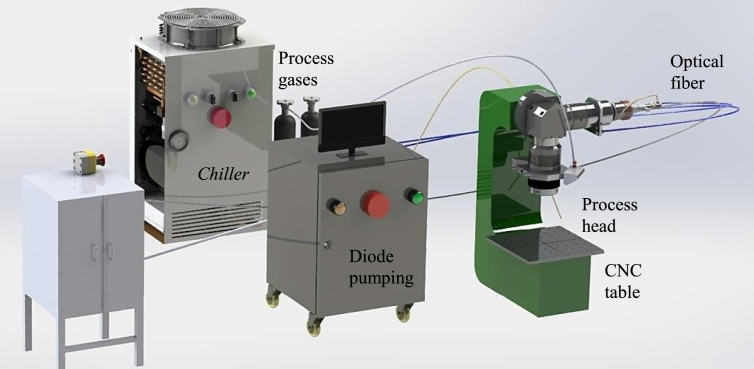
Schematic image of DedALO laser welding system.

The heat source used for welding was the Yb:Fiber laser belonging to *Laboratório Multiusuário de Desenvolvimento e Aplicações de Lasers e Óptica (DedALO),* from *Instituto de Estudos Avançados* (*IEAv – DCTA)*. An overview of the welding station, which was installed in a room with aluminum walls to confine the diffused laser beam, is shown in Figure 1. The laser equipment is the YLR-2000 model from IPG, average power 2 kW, equipped with an output fiber of diameter 50 μm and length 5 m. A coupling unit connects this output fiber to a working fiber, measuring 100 μm in diameter and 10 m in length, which is used in welding processes. The working fiber is connected to an optical collimator, composing the beam coupling system. The output beam has M^2^ quality around 9 and nearly Gaussian intensity distribution. The process auxiliary gas cylinders are positioned close to the equipment. Laser beam generation and control system can be seen in Figure 1.

The process head was coupled to an XYZ table on a milling machine, controlled by computer and driven by stepper motors, with controlled speed between 1 and 160 mm/s, X-axis range of 430 mm and Y-axis range of 508 mm, both with 5 μm resolution. A Z-axis (vertical) with 215 mm range and resolution of 1 μm was incorporated into the table.

The process head has optical assemblies made up of several elements. A first set is used to collimate the divergent beam that emerges from the optical fiber and the second is responsible for focusing the beam on the workpiece. A silica window is placed after the lenses to protect the focusing lens. The collimation and focusing optical assemblies can be exchanged to obtain different focusing distance and laser beam diameter on the workpiece. This head is tilted by 5° in relation to the normal axis, to avoid any return of radiation to the cavity, which could damage the system.

The welding table is composed of a metallic base and steel clamps with rubber tips, for sample fixation. It is possible to clamp plates up to 3 mm thick and dimensions of 100 × 200 mm.

In order to define the best process parameters for obtaining good welding results in terms of geometry and absence of discontinuities, a first set of welds was performed on the sheet surface, without filler metal, using variable welding speed and power as shown in Table 3. Beam focus was kept on plate surface, where the beam diameter is 0.1 mm. Argon was used as shielding gas, with a flow of 8 l/min. As will be seen later, new welds were produced, under the optimized conditions based on this first set analysis. It should be noted that the Zn–Fe coating layer was not removed from the sample under any of the welding conditions.

**Table 3:** Welding parameters used on first set of welds.

Condition	1	2	3	4	5	6
Speed (mm/s)	50	50	50	75	25	100
Power (W)	1500	1200	900	1200	1200	1200
Heat input (J/mm)	30	24	18	16	48	12

The welds on the optimized conditions was performed according to the following steps:

a) Sheet cutting into 100 mm × 200 mm dimensions;
b) Grinding on one side of the plate (200 mm × 1.6 mm);
c) Cleaning the plates in water with neutral soap, washing with ethyl alcohol, drying with a clean cloth followed by nitrogen blowing;
d) Assembly of two sheets on the welding table in order to form a component to be welded with a surface area of 200 mm × 200 mm;
e) Clamping of plates;
f) Carrying out the welding;
g) Clamp release;
h) Removal and weld visual examination.

### 2.3 Microstructural characterization

After laser welding, the samples were cut and prepared for metallographic analyses under an optical microscope (OM) and a scanning electron microscope (SEM). The metallographic procedures included mounting, grinding and polishing sample cross section up to 1 μm in diamond suspension, followed by etching with a 2% Nital solution, according to ASTM E407-07R15-e1.

The metallographies were obtained through a camera connected to a ZEISS/AXIOIMAGER optical microscope, model i2M, with magnification up to 1000x. The scanning electron microscope FE-SEM Tescan Mira 3 FEG, was used for microstructural analysis with magnification up to 5000x.

### 2.4 Mechanical behavior characterization

**Figure 2:**
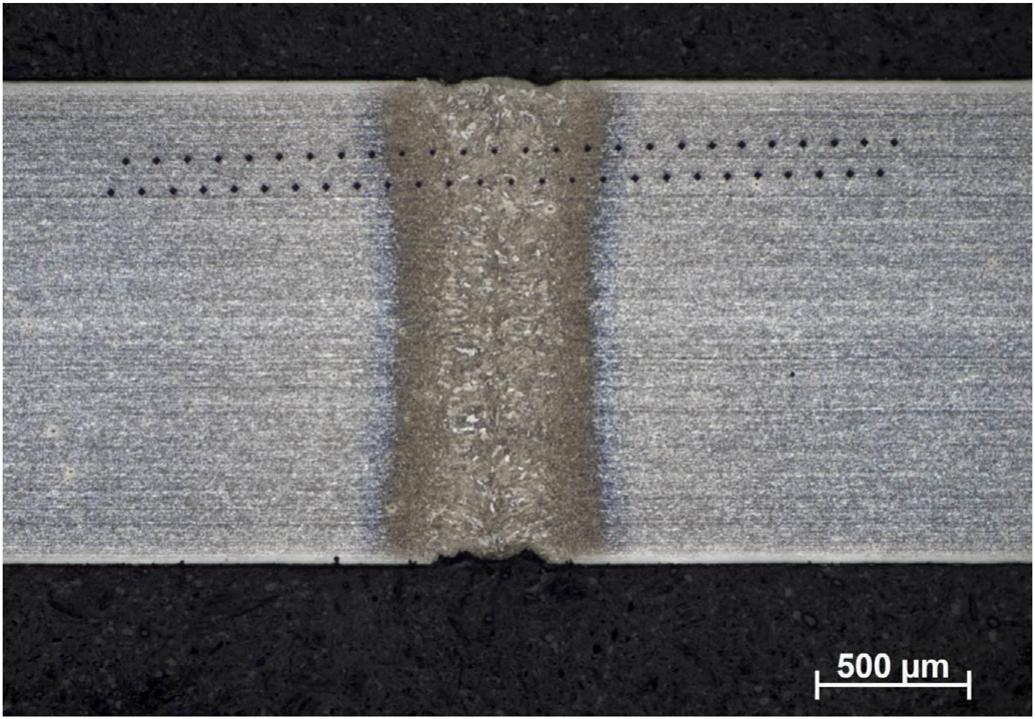
Microhardness measurements around the welded joint.

Vickers microhardness (HV) measurements were obtained using a FutureTech FM-700 microhardness tester with a square pyramidal indenter and a 136° angle between faces, applying a load of 100 gf for 10 s. A first measurement was performed on the base metal whose indentation presented straight and diagonal edges, close to 30 μm in size, so that a load of 100 gf was considered adequate. The position of the following measurements followed a "zigzag" method, moving each indentation position by 0.05 mm in the transverse direction of the weld and ±0.1 mm in the plate thickness direction, in sufficient number to ensure the coverage of fusion zone and HAZ (Figure 2). With this method, a large number of measurements is obtained when compared to measuring on a single horizontal line, without jeopardizing compliance with the requirements of ASTM E92 2017 on the minimum distance between indentations (2.5 times the average value of indentation diagonals).

Uniaxial tensile tests were performed on six samples, welded in the optimized condition (laser power 1200 W and welding speed 50 mm/s), which provided the best results, as will be discussed in the next chapter. For this purpose, the universal testing machine EMIC DL 10.000 was used, with a maximum capacity of 10,000 kgf, at a testing speed of 5 mm/min and at room temperature, in accordance with ASTM E 8M standard.

These specimens were machined in a wire EDM machine, with rolling direction kept parallel to specimen length direction. The weld bead is positioned in the center of the useable area of the specimens. Through the tests, the stress versus strain curves and the values of yield strength, ultimate tensile strength, Young's modulus, uniform elongation and maximum elongation were provided. An EE09 strain gauge, EMIC, with a measuring range from 0 to 25 mm, was used in the specimen gauge area.

The Erichsen cupping test indicates material ductility and formability through sample stretching, by means of a spherical punch that pushes the tested plate until necking or failure, obtaining a force versus deformation curve. Five specimens, welded in the optimized condition (laser power 1200 W and welding speed 50 mm/s), were tested, along with three non-welded test coupons for sake of comparison and welding effect evaluation. Those specimens were cut by electro-erosion into discs with a diameter of 35 mm, keeping the weld bead at the center of the disc for the welded samples.

**Figure 3:**
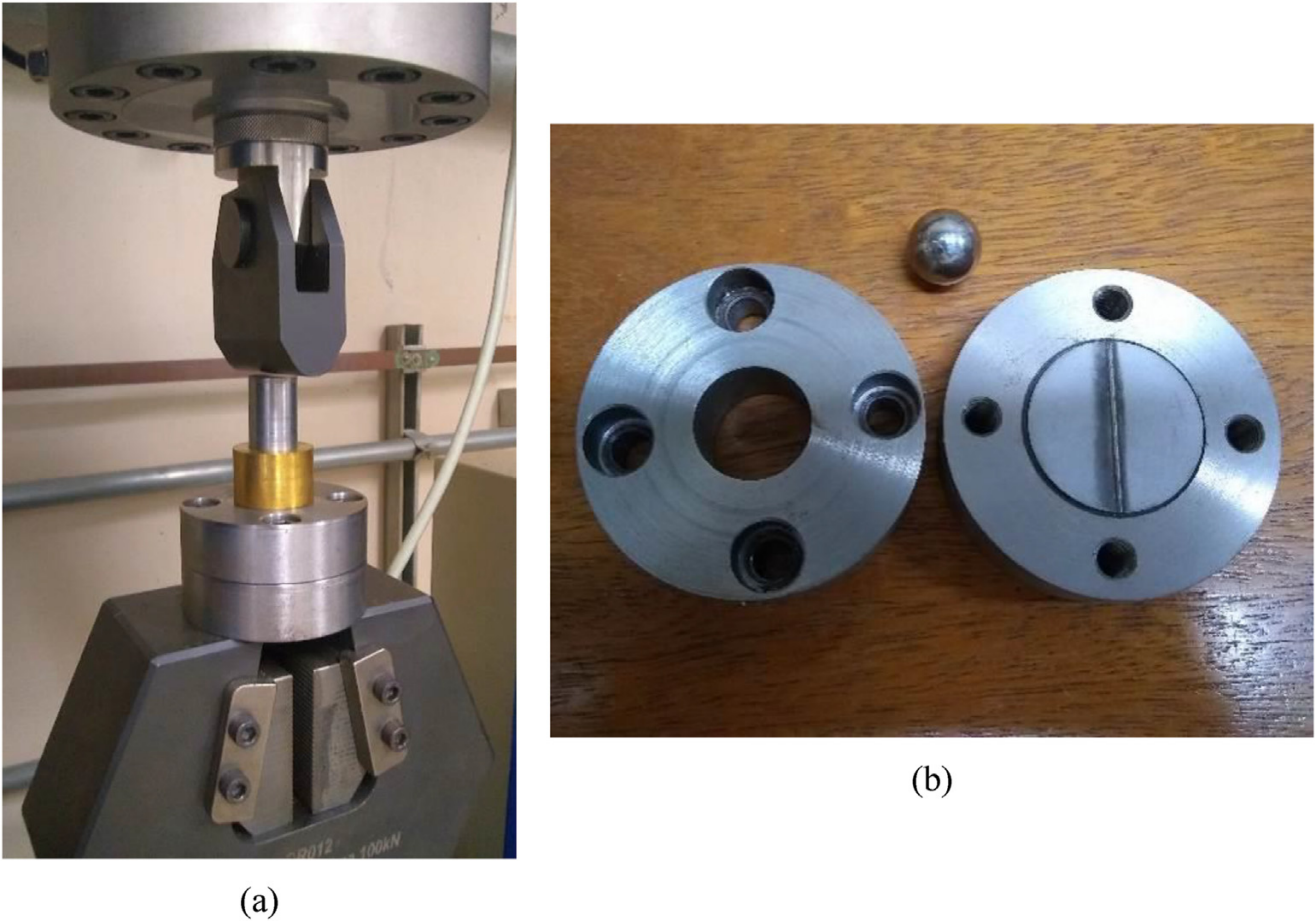
Erichsen test equipment (a), and the flange, specimen and sphere before assembly for testing (b).

A universal mechanical testing machine - EMIC, model DL10.000, was used in this test, in compression regime. The piston compression system can be seen in Figure 3a, where a sphere is compressed onto the circular plate held by a flange (Figure 3b), at a speed of 10 mm/min. The tool steel ball has a radius of 7.9 mm and was lubricated with SAE 20W-50 oil prior to testing.

The ISO 20482 standard, which standardizes this test method, defines the Erichsen index (IE) as the average of three test results of punch penetration depth into the plate. Those measurements are carried out directly at the specimen, after the test. In this work, however, the deformation at maximum load, obtained from force versus deformation curve provided by testing machine, which corresponds to the moment of specimen failure, was the measurement considered for formability analyses.

### 2.5 Welding finite element analyses

**Figure 4:**
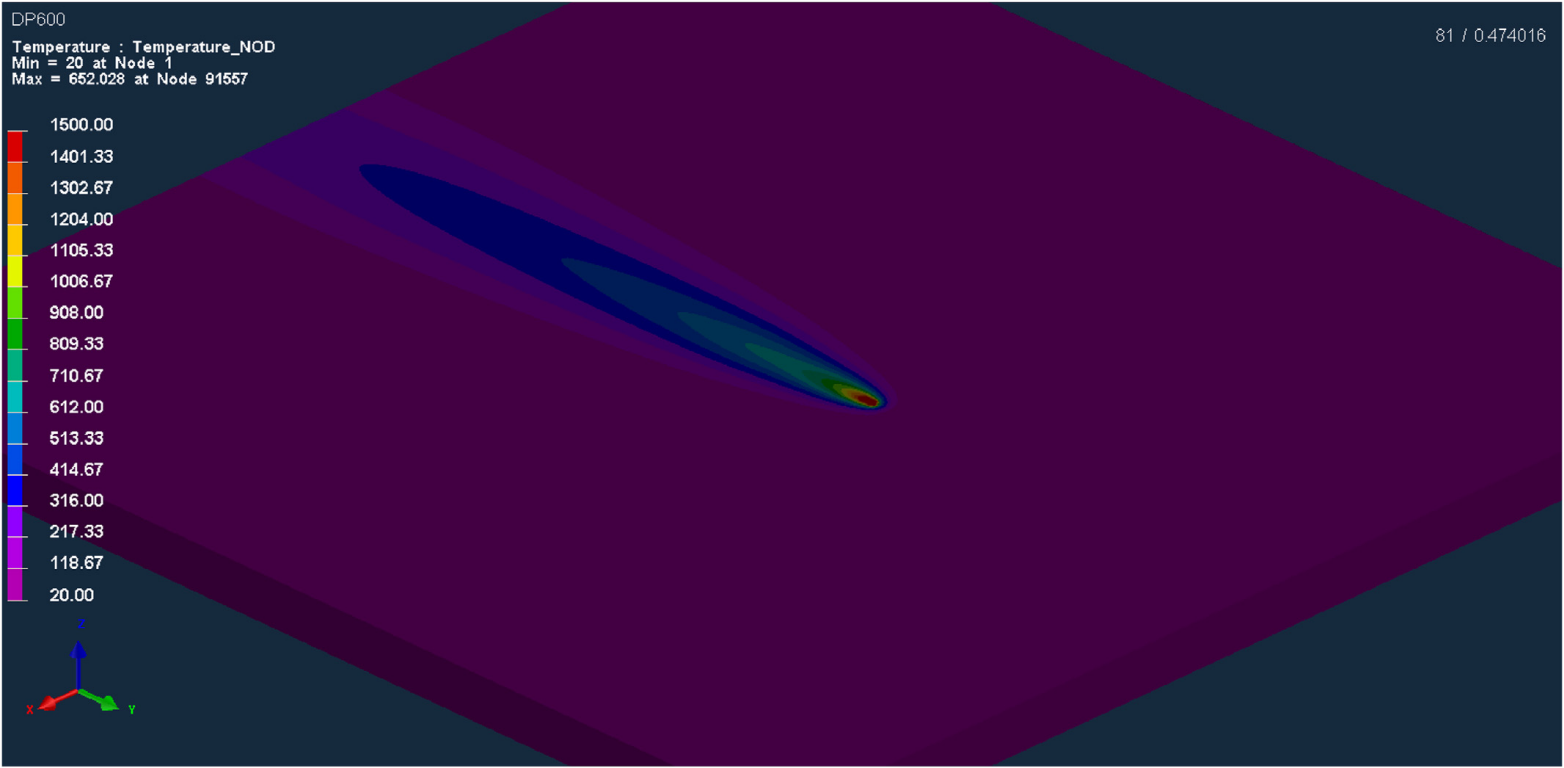
Temperature evolution during laser welding, simulated by Finite Element Analysis (FEA) on SYSWELD software.

The laser welding in the optimized condition (1200 W, 50 mm/s) was simulated by means of finite element analyses (FEA), in order to evaluate the temperature evolution throughout the welding (Figure 4). The temporal temperature evolution is hard to obtain by conventional means, due to FZ and HAZ reduced dimensions. The computerized simulation was also used to estimate residual stresses at the end of the process, creating two types of results. The first one calculates the residual stresses using the Von Mises criterion (Yang, 1980), where a positive number is always obtained. The second informs the mean stress between Von Mises, Tresca (Huang and Gao, 2004) and hydrostatic criteria, which can assume positive (tensile) or negative (compressive) values.

For the welding simulations, the software SYSWELD, from company ESI-Group (France), was used. DP 600 chemical composition, mechanical properties (yield strength, Young's modulus, poison coefficient, among others), and thermo-metallurgical properties (specific heat, thermal conductivity, density, martensitic transformation temperatures, Ac1 and Ac3) were taken from the program's embedded library.

A finite element mesh was designed to be more refined in the weld region than in the rest of the plate, in order to reduce calculation time. The simulations employed a Gaussian-type heat source that reproduces real laser beam conditions. Other variables used in actual welding, such as fixing the plate to the table (movement restrictions), ambient temperature (20 °C) and cooling speed after welding (air cooling) were respected. Other information about FEA can be obtained from the studies conducted by Bate et al. (2009) and Xu et al. (2012).

## 3 Results and discussion

### 3.1 Microstructural analyses

**Figure 5:**
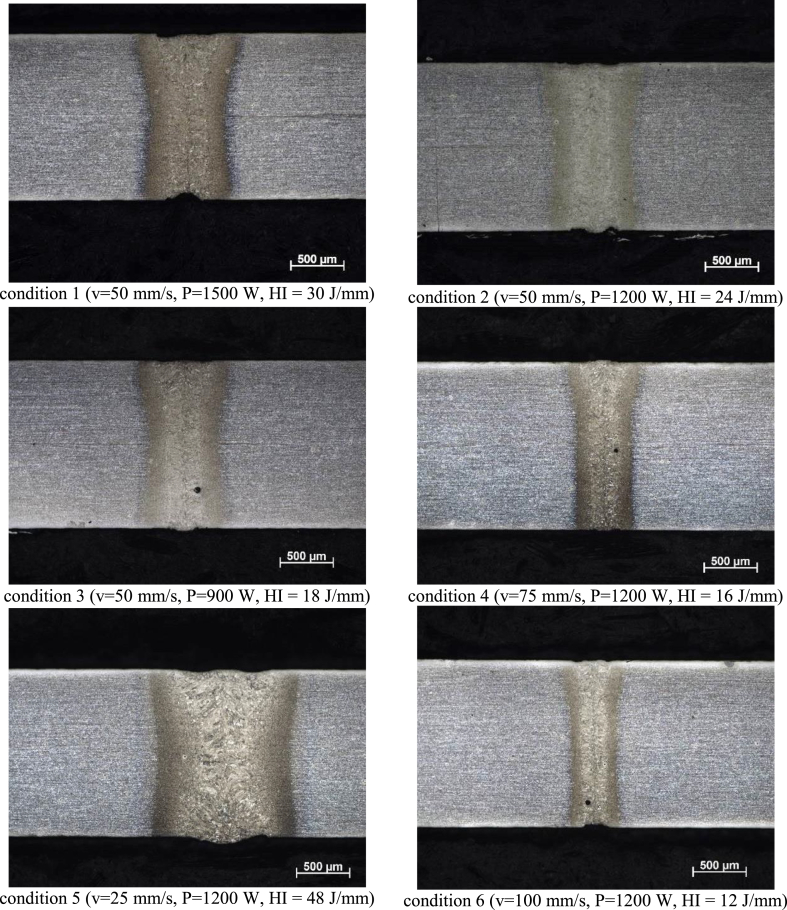
Metallographic images from transverse section of first set of samples, welded at six different conditions; etched by 2% Nital solution.

The first set of welds, performed under the six conditions presented before (Figure 5), were evaluated by optical microscope at 50X magnification, after chemical etching with 2% Nital reagent, to assess their macrostructural quality in terms of geometry and absence of defects, serving as a guide for choosing the best welding condition for subsequent studies. Figure 5 presents these images arranged side by side for comparison. The microstructural features were not analyzed at this first time.

The first aspect to be observed is concerning the weld geometry. An inversely proportional relationship between welding speed and weld bead width can be clearly seen. A directly proportional relationship between weld power and width can also be observed, although this is not as evident as the influence of speed. These observations can be attributed to the fact that a lower speed increases heat input (HI) as well as heat dissipation in the direction transverse to the weld, which in turn increases the region that will exceed critical temperature for phase transformation during welding. Conversely, a higher welding speed provides less heat input and lead narrower temperature profiles and, consequently, narrower weld beads. Laser beam power presented an inverse tendency to that of speed, that is, a higher power leads means more heat input and, consequently, a wider weld, for the same reasons mentioned above (Alves et al., 2018).

Figure 5 also allows us to observe that all welding conditions provided full penetration melt depth along material thickness, and no cracks were visible. However, condition 5 (highest heat input used in the study) resulted in an excessively wide weld when compared to the other conditions, which could be detrimental to mechanical properties, both by obtaining a less refined microstructure and by the possibility of martensite softening at HAZ (Xie et al., 2017).

Conditions 4 and 6 (lowest heat inputs) resulted in very narrow welds, whose characterization would be difficult to carry out. Furthermore, narrower welds require much more accurate control of the laser beam position over the joint, eventually leading into inconsistent welds.

The welds produced under conditions 3, 4 and 6, the presence of small pores was noticed, possibly caused by keyhole instability, which depends on a delicate balance between metal evaporation, hydrostatic pressure and surface tension inside the weld pool (Matsunawa et al., 2003). During welding, small keyhole disturbances cause instability in the liquid metal wall, generating bubbles. If those bubbles do not migrate to the welding surface and are expelled, they will be trapped into weld pool, turning into porosity after weld solidification (Katayama et al., 2010).

The fact that three welds were free of porosity indicate that laser welding without Fe–Zn coating removal did not cause pores. These results contradict those of Zdravecká and Slota (2019) and Mei et al. (2009), which show that Zn has a preponderant effect on porosity formation at FZ. One of the possible explanations is that the mentioned authors employed laser with CO_2_ resonator, instead of fiber laser as in the present study. Fiber laser beam quality, wavelength and intensity allow eliminating the Zn–Fe layer before it contaminates FZ liquid metal, as already published by other works from DedALO laboratory (Ladario et al., 2007).

The test specimen of welding condition 1, in turn, presented some loss of material, comparatively more intense than that of condition 2, which can be attributed to the use of the highest laser power among all test conditions.

Due to the aspects listed above, considering weld bead morphology, the presence of discontinuities and the ease of characterization, condition 2 (v = 50 mm/s and P = 1200 W) was considered the most appropriate for the welding of this material. Thus, welded samples for all the following tests (metallographic analysis, Vickers microhardness, uniaxial tensile tests, Erichsen cupping tests) will be carried out according to this condition.

**Figure 6:**
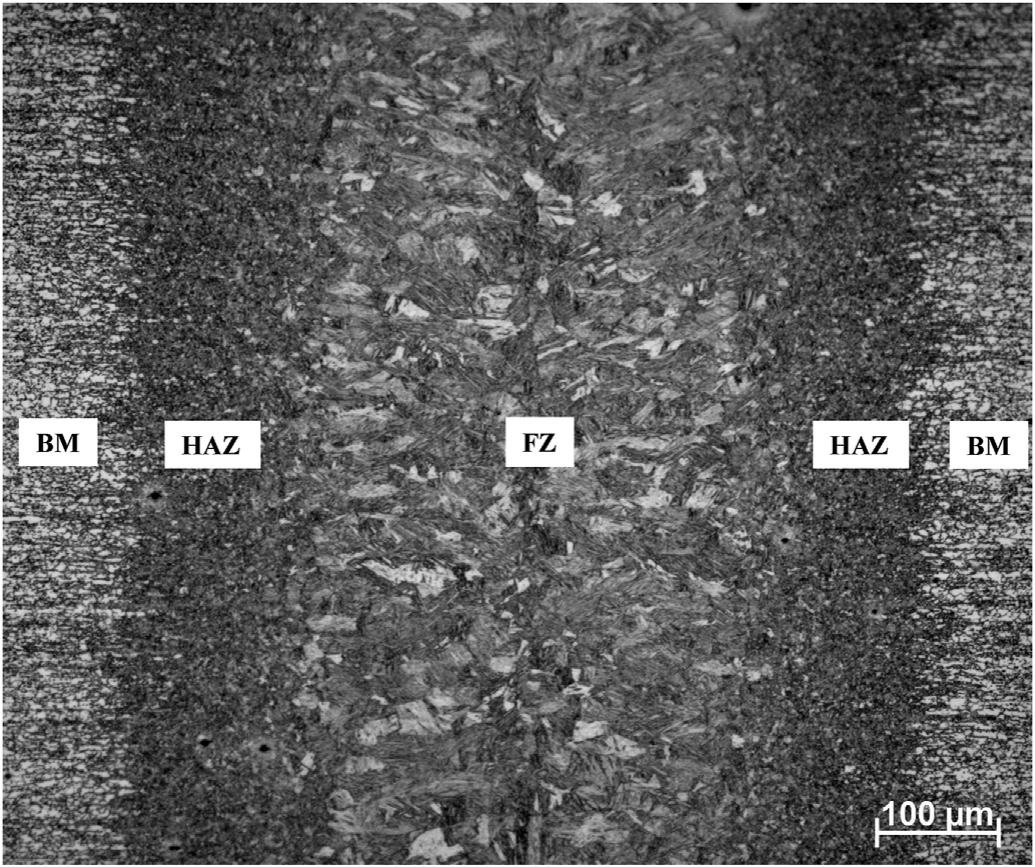
Macrographic image of weld performed at 50 mm/s and 1200 W (condition 2).

The macrographic characterization of a weld performed in condition 2, obtained with optical microscope and 2% Nital etching, is shown in Figure 6. Three different regions can be distinguished, as discussed below.

The grain-decorated area at the center of the image constitutes the Fusion Zone (FZ), in which the material was melted during the welding processes. In this region, a coarser microstructure with some evidence of columnar grains can be observed, promoted by epitaxial growth from non-melted grains.

On both sides of the weld, at each edge of the image, the original base metal (BM) is visible. On those areas, the applied heat was not sufficient for the occurrence of visible phase transformations, and it is even possible to observe a typical rolling texture of the material in the as supplied condition.

Between FZ and BM, the two bands that separate these regions locate the Heat Affected Zone (HAZ). Peak temperatures achieved at HAZ during welding, although insufficient for fusion, led to total or partial microstructural phase transformations. The grain structure in HAZ seems to be more refined than BM and FZ.

It can be seen in Figure 6 that the weld obtained under the optimized condition 2 presented relatively constant width along the thickness, which is a characteristic of keyhole laser welding with full penetration. FZ width was measured as around 0.3 mm in the middle of the bead, whereas HAZ presented parallel geometry in relation to weld bead and width of approximately 0.1 mm on each side.

**Figure 7:**
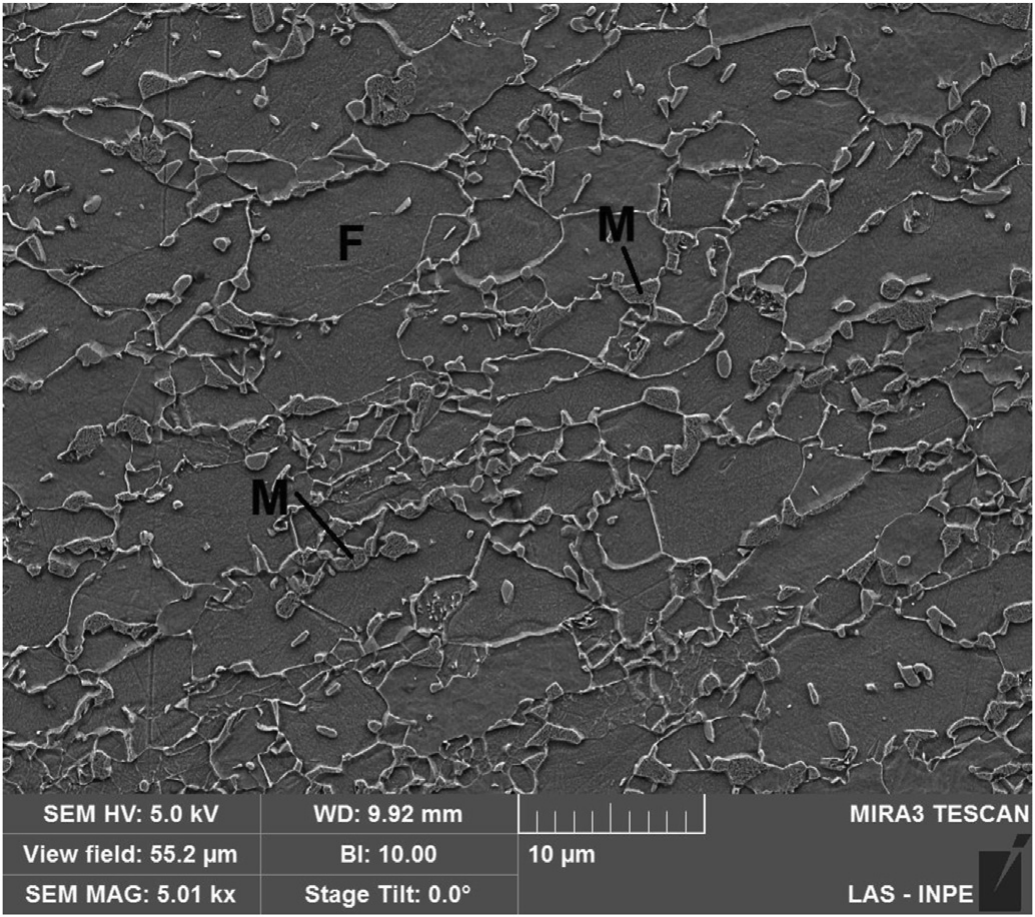
Base metal microstructure in the “as-supplied” condition, composed of a ferritic matrix (F) and disperse martensite islands (M). SE-SEM.

These three weld regions were observed in detail through Scanning Electron Microscope (SEM) images. Figure 7 shows SEM secondary electrons (SE) image from base metal microstructure, promoted by the sheet metal fabrication process (hot and cold rolling followed by intercritical annealing). This microstructure is basically constituted by a ductile ferritic matrix, complemented by martensite islands that contribute to increase the strength of the material. The volumetric fraction of martensite was not measured, but studies on similar DP steel grades indicated amounts around 20% in the as-supplied state (Balbi et al., 2018; Correard et al., 2016). Another evident characteristic is that martensite islands are dispersed in the matrix of ferritic grains; this is due to the fact that martensitic transformation originates from austenite grains, which, in turn, are generated from the pre-existing ferrite-pearlite structure during intercritical annealing (Das and Chattopadhyay, 2009; Kalhor et al., 2020).

**Figure 8:**
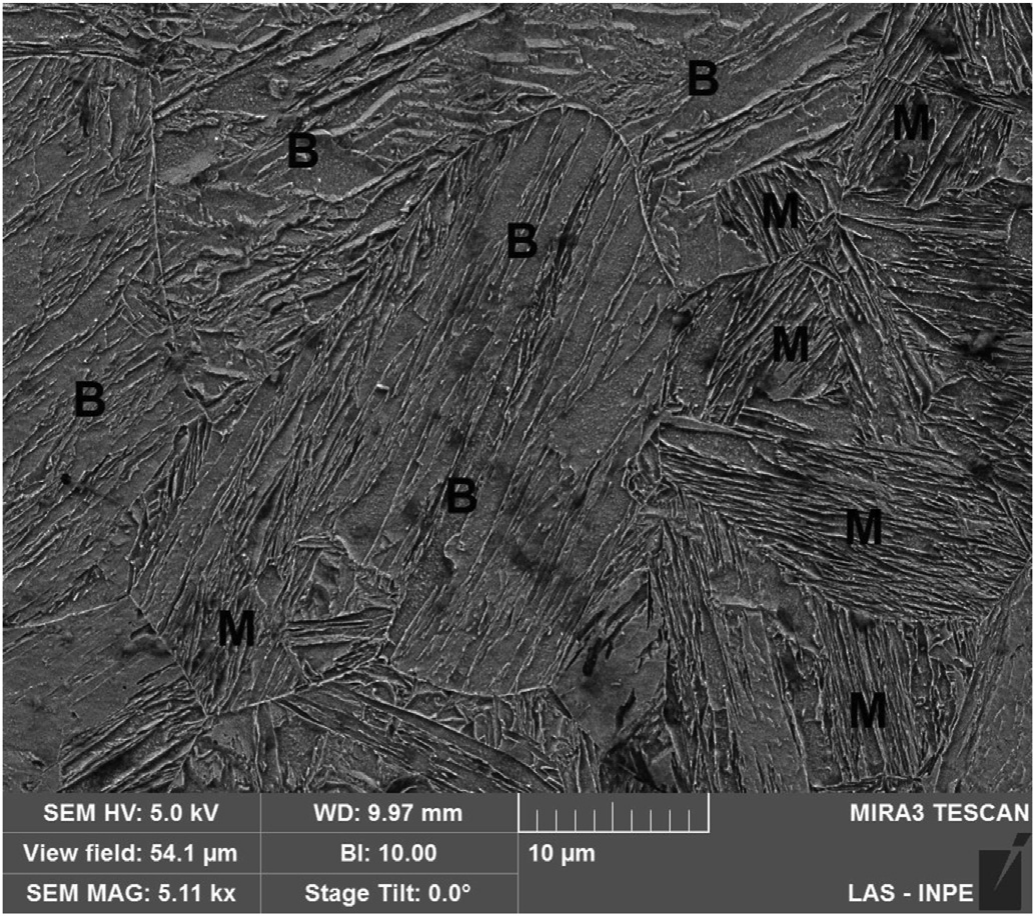
FZ microstructure, composed of bainite (B) and martensite (M).

The Fusion Zone can be seen in more details in Figure 8. Due to the high heating and cooling rates imposed in this region during welding, a microstructure composed of bainite and martensite was obtained, with much higher hardness levels that those observed on base metal, as will be discussed later. In the image, grains with more refined lath structure were identified as martensite (M), while those with less evident laths and some incipient indication of carbide coalescence were characterized as bainite (B). For a more accurate classification, however, it would be necessary to characterize those samples in a transmission electron microscope (TEM). It is noteworthy that other studies have already indicated the presence of bainite at FZ welds in DP steels (Alves et al., 2018; Correard et al., 2016).

**Figure 9:**
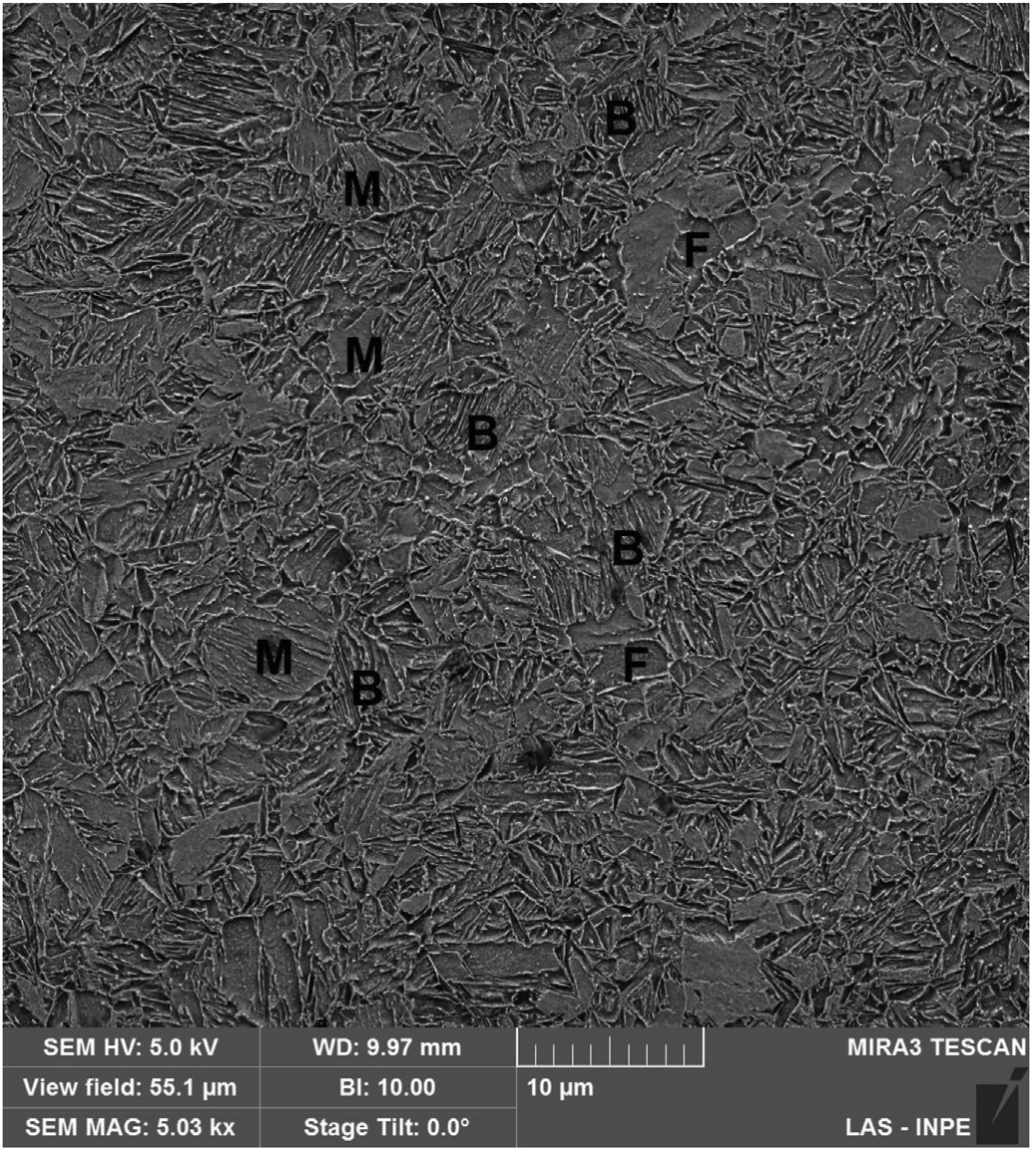
HAZ microstructure, composed of bainite (B), ferrite (F) and martensite (M).

The Heat Affected Zone (HAZ) presents a combination of bainite, martensite and ferrite features (Figure 9). HAZ that are closer to FZ experienced relatively high temperatures imposed by welding, although under melting point (*liquidus* Fe–C diagram line), being subjected to full austenitization and subsequent formation of a predominantly bainitic/martensitic structure.

The subsequent HAZ area, not so close to the weld, constitutes the intercritical region, where temperatures were not sufficient to exceed the F + A region, so that other microconstituents are expected. In this region, as we can see in Figure 9, it is already possible to observe some ferrite grains (F), besides martensite (M) and bainite (B). The ferrite comes from the original base metal, due to the temperature between Ac1 and Ac3, or due to the short residence time at these temperatures. Naturally, moving away from weld fusion line in the transverse direction, there are regions that underwent lower peak temperatures and cooling rates during welding, leading to a gradual increase in the fraction of untransformed phases (ferrite and martensite) until the end of HAZ, where the phase balance corresponds to the expected fractions for the base metal. Last, there is no evidence of a tempered martensite zone, where one would expect a decrease in hardness.

By comparing HAZ and FZ, it is possible to verify that the latter presents a much coarser microstructure, compatible with phase nucleation from liquid state. HAZ transformations, on the other hand, occur in the solid state and, by having a much larger number of available interfaces for nucleation of new grains, the resulting microstructure tends to be more refined and complex (Alibeyki et al., 2018).

### 3.2 Vickers microhardness

**Figure 10:**
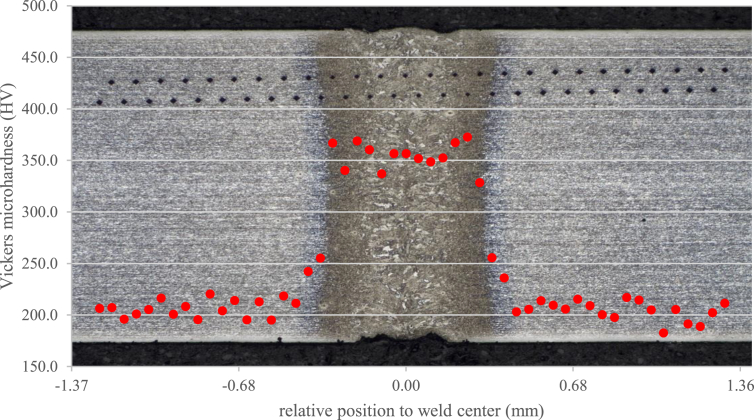
Hardness profile of weld performed on condition 2, compared to actual weld macrographic image.

The microhardness measurement results on a welded sample can be seen in Figure 10. Those results were carefully superimposed on the macrographic image, allowing comparison between the hardness values and the different weld regions.

Hardness is remarkably high in FZ and in HAZ adjacent to fusion line (FL, i.e. the interface between FZ and HAZ), reaching values exceeding 350 HV. The hardness increment can be explained by the fact that those regions are predominantly composed of bainite and martensite, as a result of the high cooling rates imposed by welding, as mentioned above. It can also be observed that HAZ region adjacent to FL presents hardness values slightly higher than those found at FZ, which can be attributed to the more refined microstructure in the former.

On the other HAZ hardness values start to decrease as we move away from the weld, in the direction of thermal gradient, as a function of the increasing ferrite fraction which is a softer phase. This trend is observed until hardness values match with the ones obtained at base metal (approximately 200 HV). These values are in accordance with those reported in the literature for this material and welding process (Dong et al., 2014; Evin and Tomáš, 2017; Xie et al., 2017), indicating weld bead hardening over 60% from the BM.

It is clear from Figure 10 that HAZ softening, caused by base metal martensite tempering during welding, was not observed in the present study. This phenomenon could lead to HAZ hardness values lower than those obtained at base metal. Based on studies by Zhao et al., 2013, two factors can explain this finding:

a) Softening is directly proportional to the grade of steel. As DP600 steel contains, in the “as-supplied” condition, a relatively low volume fraction of martensite (around 20%), tempering would affect a quite small fraction of the material.
b) Tempering time is proportional to the inverse square of welding speed. The reduced HAZ dimensions (around 0.1 mm) is this welded part indicate a relatively high welding speed, inducing phase transformations in a very narrow region. This fact suggests that there was not enough time for martensite tempering at HAZ subcritical region, in a level that could affect its hardness.

### 3.3 Uniaxial tensile tests

**Figure 11:**
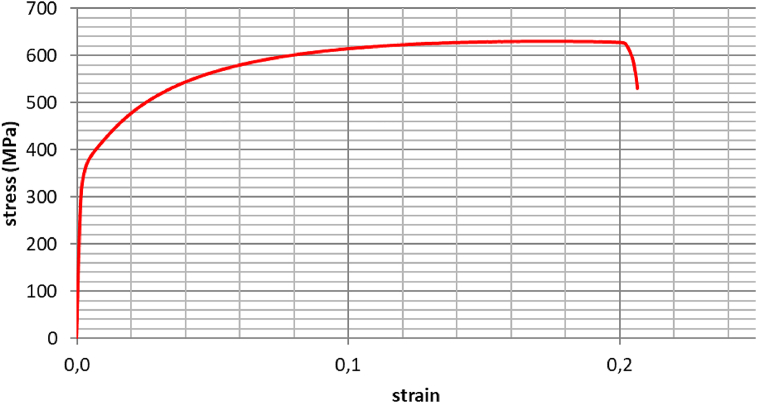
Stress *versus* strain curve, obtained by uniaxial tensile test on a welded sample.

Uniaxial tensile tests of laser-welded joints presented typical engineering stress x strain curves, without a defined yield point, as can be seen in Figure 11, which presents the results of one of the samples, for illustrative purposes.

The continuous yield limit is a characteristic of dual phase steels. It is promoted by the large number of high mobility dislocations, generated by the volumetric expansion of the martensitic transformation, which facilitates the onset of plastic deformation (Ghaheri et al., 2014; Habibi et al., 2016; Maleki et al., 2018; Tian et al., 2020). This characteristic can be changed by laser welding, though, if the heat input is sufficient to promote diffusion of interstitial atoms such as carbon and nitrogen to the core of those dislocations, creating a pinning effect that restrain their mobility and thus generating a yield range discontinuity (Farabi et al., 2010). In the present study, the absence of a discontinuous yield indicates that the heat input was not enough to stimulate this atomic movement, in a level that could interfere with material mechanical behaviour in this aspect.

The overall tensile test results on welded specimens can be viewed on Table 4. The material data in the “as-supplied” condition (without any welds), extracted from steel maker certificate, were added to the table for comparison, since no tensile tests were carried out in non-welded samples. It is clear from the results that, considering the statistical variation, yield and tensile strength are pretty much the same on welded and non-welded materials. This observation corroborates the previously mentioned fact that welding methodology used in this study did not change the component yield behaviour. All samples reached minimum required Ultimate Tensile Strength (UTS) for DP600 classification (600 MPa). The measured values of the Elastic Modulus, or Young's Modulus, also were within expected range for this material (Wu-Rong et al., 2011). The measured Young's Modulus (E) of the current Usiminas' laser welded blanks (220 GPa) is an original contribution of this work.

**Table 4:** Uniaxial tensile test results for welded samples.

Sample	Yield Strength (YS) (MPa)	Ultimate Tensile Strength (UTS) (MPa)	Uniform elongation (ε_u_) (%)	Maximum elongation (ε_m_) (%)	Young's Modulus (E) (GPa)
1	373	636	15	21	220
2	349	622	15	22	228
3	371	631	17	23	201
4	362	626	15	22	205
5	355	630	16	24	278
6	370	628	16	17	200
Mean/Standard Deviation	363 ± 10	629 ± 5	15,7 ± 0,8	21,5 ± 2,4	222 ± 30
“as-supplied” material data	350 ± 4	635 ± 4	19,5 ± 0,5	29,0 ± 0,8	∗

∗ not informed.

The similarity between welded and base metal samples, regarding yield and ultimate tensile strength, combined with the fact that all tensile test specimens broken at base metal, far from weld HAZ, confirm the absence of softening phenomenon, in good agreement with microhardness test results. It is well known that base metal martensite tempering, promoted by welding, can lead to a localized hardness decline at subcritical HAZ. This softened region creates a preferential failure spot during tensile tests, in which yield would occur at lower-than-expected stress level. HAZ softening weakens the welded component and damages its future operation (Gao et al., 2018; Wang et al., 2016).

The average reduction of roughly 4 percentage points in uniform elongation of welded samples, when compared to original material, can be attributed to the weld bead present at the center of the specimen which, due to its large volumetric fraction of bainite and martensite, constitutes a hardened region that does not deform as the rest of the sample. This strain discontinuity had been reported before (Fernandes et al., 2017) and always implies in depletion of perceived ductility at tensile test welded specimens. Indeed, the welds did not presented visible necking, unlike the rest of the specimen, which had its transverse section visibly diminished. The observed uniform elongation reduction cause by welding does not necessarily indicate loss of material formability, unlike findings by Panda et al. (2008). Erichsen cupping tests, which will be seen next, are best suited for this evaluation.

### 3.4 Erichsen cupping tests

As presented before, the presence of bainite and martensite at weld bead creates a hardened region of reduced elongation. In order to evaluate its influence on ductility and formability of the material, Erichsen cupping tests were carried out. These tests do indicate the behaviour of a DP600 steel sheet, containing a central welded joint, under cold stamping conditions.

The Erichsen test results on welded specimens are shown in Table 5. Three samples without weld were also tested, for the sake of comparison. The Maximum Force (Fm) and Ultimate Collapse Force (UCF) values for the two kinds of samples (base metal and welded) are similar. The punch total penetration, represented by deformation at maximum load as taken from testing machine force *versus* deformation curve, presented minor reduction (7.1%), from base metal to welded samples.

**Table 5:** Erichsen cupping test results.

#	Type	F_m_ (N)	UCF (N)	Deformation at maximum load (mm)
1	**Base metal (no weld)**	47552	42797	14.28
2		44996	40497	14.69
3		46655	41990	15.00
**Mean**		**46401**	**41761**	**14.66**
**Standard deviation**		**1297**	**1167**	**0.36**
6	**Welded samples**	46882	42194	14.66
7		47610	42849	14.53
8		45381	40843	13.69
9		45525	40973	12.45
10		46899	45509	12.77
**Mean**		**46459**	**42474**	**13.62**
**Standard deviation**		**966**	**1894**	**1.00**

These results are similar to those found by Wang et al. (2017), indicating that hardening promoted by a laser beam, according to welding parameters described in this work, did not cause significant problems on laser welded DP600 steel sheets forming. The absence of HAZ softening also contributes positively to formability, since the softened region tends to undergo a thickness reduction (necking) at early stages of deformation, leading to premature sheet rupture during forming (Wang et al., 2017).

### 3.5 Numeric simulations

This section describes the results of laser welding numeric simulations, based on Finite Element Analysis (FEA), performed in SYSWELD® software. Welding was simulated for the optimized condition, as defined on section 0 (i.e. v = 50 mm/s and P = 1200 W).

**Figure 12:**
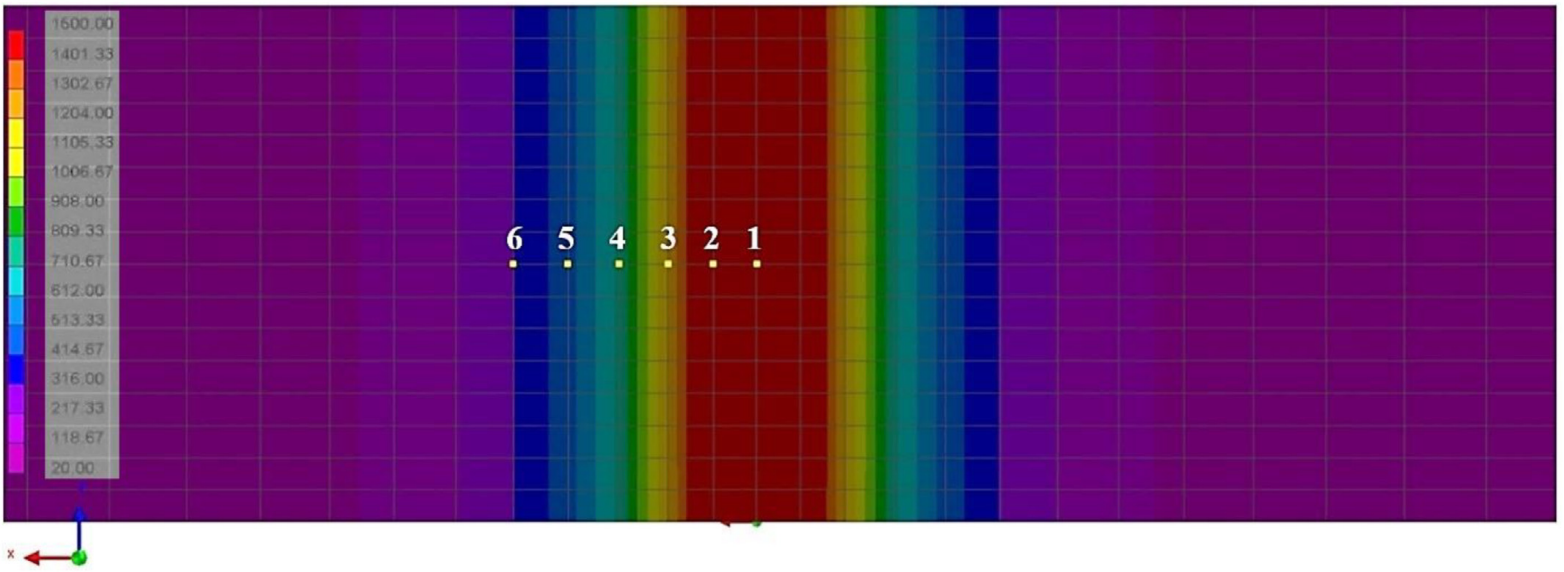
Simulated temperature profile (°C) on weld transverse section, in a specific time.

Figure 12 shows the simulated temperature profile, in a cross section of the sheets in the weld region. It is noteworthy that this thermal profile is related to a specific temporal shot, not representing the peak temperatures in each region, as these are reached at different times, as will be seen below. As expected, the temperatures in the center of the weld reach the highest levels, exceeding the melting point of the material, and decrease as one move towards the base metal. The shape of the weld in the transverse section was simulated by the program as a straight geometry, which is compatible with the macrographs observed in this work and keyhole laser welds in general (Figure 6).

**Figure 13:**
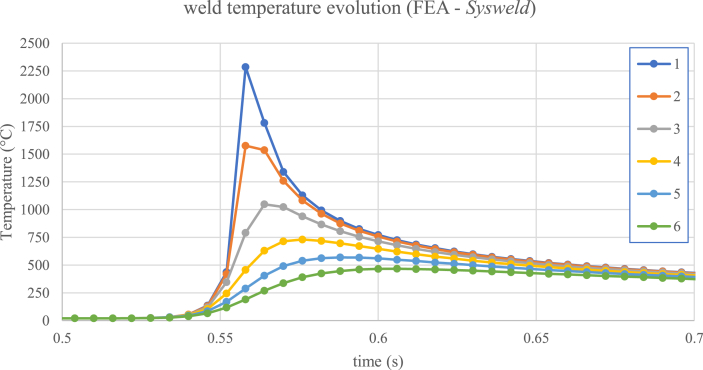
Temperature evolution with time, simulated on six different points of the weld, as indicated in Figure 12.

The simulation of temperature evolution over time, for each of the 6 points indicated above on Figure 12, can be seen in Figure 13. By looking at the time scale (X-axis), in tenths of seconds, the energy concentration of laser welding heat source becomes evident, generating extremely high heating and cooling rates. It can also be seen that the maximum temperatures at each point are reached at slightly different times.

The temperature curves indicate that only points 1 and 2 exceeded the steel melting point, reaching temperatures above 1500 °C, thus delimiting the fusion zone (FZ). Temperature drops after reaching the peak is abrupt, particularly at point 1 where cooling rate exceeds 80,000 °C/s. Point 3 reaches temperatures over 1000 °C, substantially above Ac3 (867 °C, according to SYSWELD database), indicating total austenitization in this region, but the cooling rate at this point is lower than at points 1 and 2. Point 4 reaches temperature Ac1, defined by program database as 727 °C, above which partial austenitization starts to occur, but the residence time above Ac1 is very short (less than 0.01 s). Thus, it can be said that HAZ, according to simulated data, is comprised in the region between points 2 and 4. At points 5 and 6, temperatures remain below 600 °C, so that no phase transformation is expected beyond point 4.

In short, the different peak temperatures and cooling rates promoted by laser welding along weld cross section, clearly illustrated by the simulations, are compatible with the variety of microstructures observed in metallographic tests.

Based on the simulated thermal cycles and material properties, the numerical simulation also estimated the proportion of phases present after weld completion. The program database for DP600 steel foresees the following phases: initial material, filler metal (nonexistent in this case), martensite and austenite. Due to the high cooling rates, only the martensite transformation is considered during cooling. It should be noted, however, that the fraction of martensite indicated by the simulation includes only martensite transformed during welding, not including the pre-existing martensite from base metal. The other microconstituent present, complementary to martensite, is the initial material (ferrite plus martensite), equivalent to the material in the supplied state, composed of ferrite and martensite.

**Figure 14:**
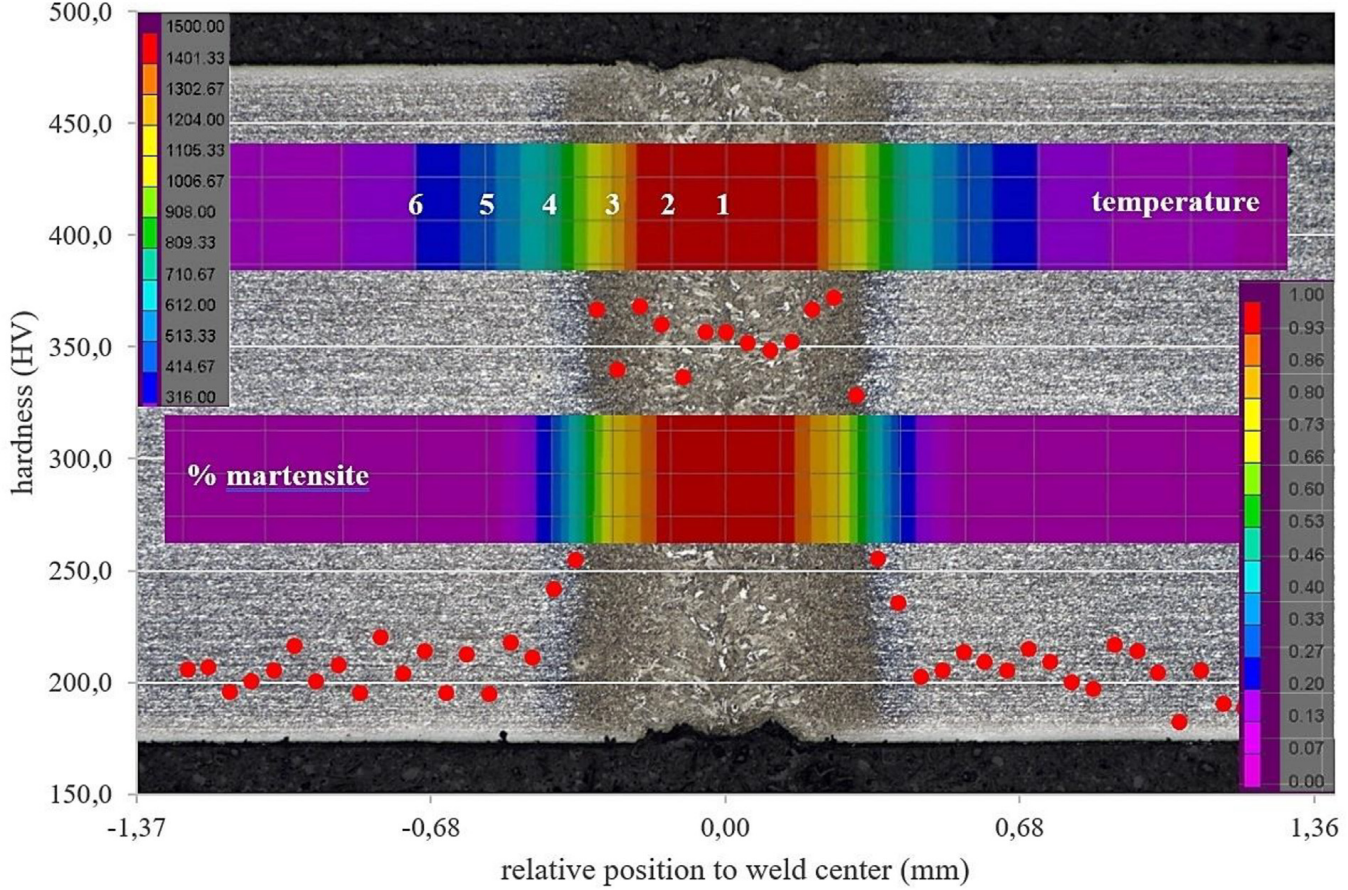
Comparison of real microstructure and hardness values with temperature profile and estimation of martensite fraction, as provided by numerical simulation.

The results of this simulation are shown in Figure 14 color pattern, which exhibits the proportion of martensite formed during welding. This proportion reaches 1 (100%) at FZ, decreasing gradually as one moves away from the weld until reaching approximately 20% in the base metal. These estimations are consistent with metallographic test observations, considering simulation restriction in the sense of disregarding the bainitic transformation. Figure 14 also brings again microhardness test results, this time superimposed by the temperature simulations and martensite proportion. All the three images are in the same scale, in order to allow their comparison. Care was taken to keep all hardness measurements visible in the weld area. It is clear from the image composition that numerical simulations performed by SYSWELD achived excellent agreement with real weld microstructure and microhardness values. For instance, it can be observed that the simulated temperatures close to Ac1 (point 4) reasonably correspond to the end of both HAZ and the martensitic transformation, while also matching with the region where hardness values reach base metal levels. Comparison between macrographic image and temperature simulation shows that FZ limits converge with point 2, which is very close to melting temperature, and with the 100% martensitic region indicated by the phase proportion simulation. The fraction of martensite shown in Figure 14 is only the one that was transformed after the welding. The initial material on both sides of the central colored band in Figure 14 should represent a balance between martensite and ferrite. However, one must understand only the fraction transformed in the central region and the phase balance in HAZ and BM are incorrect in view of the DP600 base material (Figure 7).

**Figure 15:**
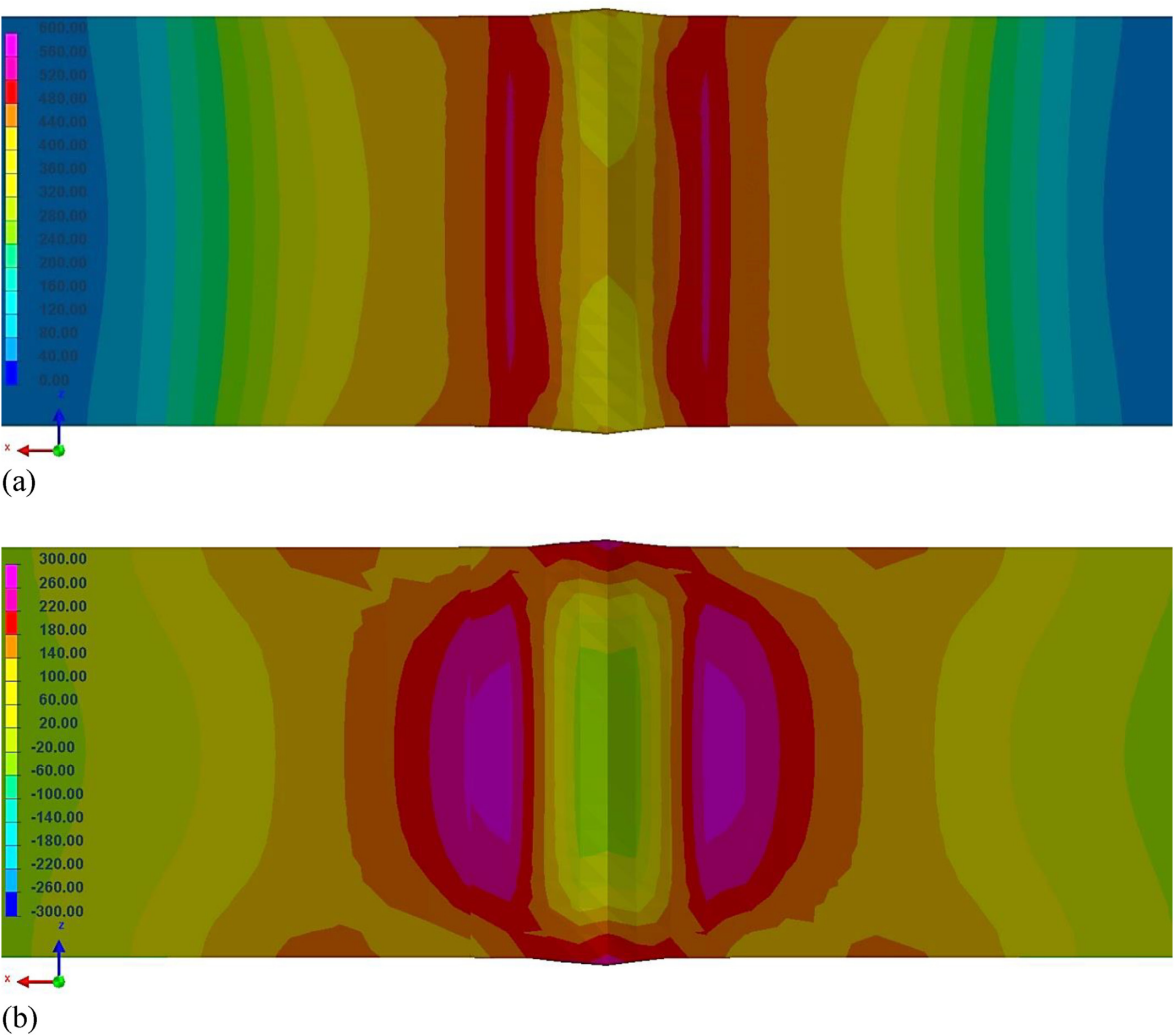
Residual stresses simulated in a weld cross section: (a) Von Mises criterion and (b) mean values.

Considering the excellent correlation between simulation and actual weld characteristics, computer simulations were also used to estimate the level of residual stresses and distortions induced by laser welding on DP600 sheet. Figure 15a shows the residual stresses according to Von Mises criterion in a cross section of the weld. These stresses reach levels above material's yield strength, whose average value obtained in this work was 363 MPa, especially in a narrow region between FZ and HAZ where they exceed 500 MPa. This observation does not necessarily indicate that welding generates plastic deformation in the weld region, as the martensitic microconstituent has a much higher strength than that of the base material (Krauss, 1999). At welded joint central region, stress levels are lower, which can be attributed to the martensitic transformation that is associated with a volumetric expansion (Tian et al., 2020).

Figure 15b shows the estimated mean residual stresses (average between Von Mises, Tresca and hydrostatic pressure models). The maximum values were also found at the interface between FZ and HAZ but, in comparison to Von Mises criterion, stress levels are lower, not exceeding 300 MPa. This simulation indicated slightly compressive residual stresses at the center of the weld bead. Unlike the Von Mises criterion, mean stress can present negative values, which indicate the presence of compressive stresses, such as those observed in this case.

**Figure 16:**
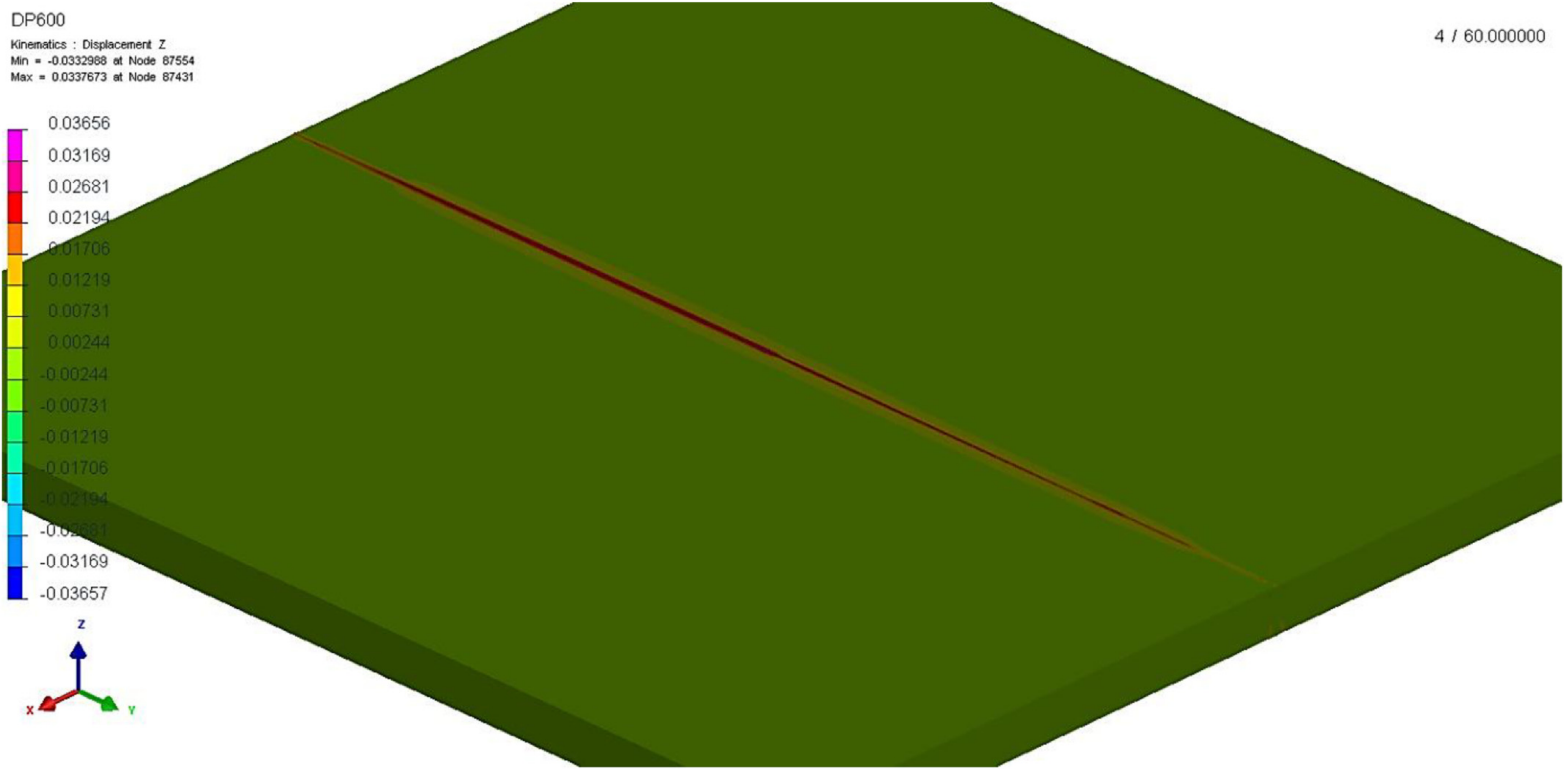
Vertical displacement simulation.

Figure 16 illustrates the simulation results for vertical displacement (deformation on the Z-axis, coplanar to plate upper surface), which indicates possible distortions caused by welding. Although the simulation indicated high residual stresses at welded region, simulated strain values are quite low, not exceeding 22 μm at the center of the plate. This information is compatible with the experimental findings, as no visible distortions were observed in any of the welded specimens. The absence of distortion and warping in the welded joint is one of the great advantages of laser welding, when compared to arc processes.

## 4 Conclusions

A first set of six bead-on-plate welds was generated to define best welding parameters for subsequent tests. By taking into account the weld bead morphology, the absence of discontinuities and the ease of characterization, condition 2 (laser power 1200W, welding speed 50 mm/s) was considered the most appropriate for the welding of this material. Thus, a second set of butt joint welded samples, for metallographic analysis, Vickers microhardness, uniaxial tensile tests, and Erichsen cupping tests, were produced according to this condition.

The welded joint obtained under the optimized condition presented relatively constant width along the thickness, characteristic of full penetration keyhole laser welding. FZ width was measured around 0.3 mm in the middle of the bead, whereas HAZ presented parallel geometry in relation to weld bead and width of approximately 0.1 mm on each side.

The weld microstructure was characterized as martensite plus bainite in the fusion zone (FZ), with growing fractions of ferrite at Heat Affected Zone (HAZ) as one moves away from fusion line until base metal (BM), which exhibits DP600 typical microstructure composed of martensite islands surrounded by a ferritic matrix. Hardness is around 60% higher at FZ than at BM, being maximum at supercritical HAZ due to its highly refined microstructure and decreasing with the increase of ferrite amount from FZ to BM, until reaching original hardness levels. HAZ softening due to martensite tempering, that could weaken the joint, was not observed.

Uniaxial tensile tests were performed on six welded samples and results were compared with reference data from steel manufacturer for “as-supplied” condition. Results presented typical engineering stress x strain curves, without a defined yield point. Yield and tensile strength on welded samples were similar to base metal values, reaching the required values for DP600 classification (minimum tensile strength 600 MPa). The similarity between welded samples and base metal reference, combined with the fact that all tensile test specimens ruptured far from HAZ, confirm the absence of softening. The average reduction of 4 percentage points in uniform elongation of welded samples, when compared to original material, can be attributed to the hardened weld bead present at the center of the specimen that does not deform as the rest of the sample.

Erichsen cupping test were performed on five welded and three non-welded specimens. Maximum force (Fm) and Ultimate Collapse Force (UCF) values for the two kinds of samples are statistically similar. Punch total penetration, represented by deformation at maximum load, presented minor reduction (7.1%) on welded samples. These results, along with absence of softening mechanism, indicated that laser welding, according to parameters set in this work, did not cause significant problems that could harm future material processing.

Numeric simulations based on Finite Element Analysis (FEA) were carried out to estimate temperature evolution, phase proportions, residual stresses and distortion levels. Excellent agreement with actual weld macrographic images and microhardness profile was obtained. Despite high estimated residual stress levels, simulated distortions were considerably low (22 μm maximum), compatible with the absence of visible distortion in all welded specimens.

Based on experimental and simulated test results, fiber laser welding applicability in Dual Phase 600 1.6 mm thick steel sheets, according to optimized parameters defined in this work, was considered suitable for industrial application, being not detrimental to subsequent forming operations and future use of welded components.

## Declarations

### Author contribution statement

Vinicius Machado Mansur: Conceived and designed the experiments; Performed the experiments.

Raquel Alvim de Figueiredo Mansur: Performed the experiments; Contributed reagents, materials, analysis tools or data.

Sheila Medeiros de Carvalho: Performed the experiments; Wrote the paper.

Rafael Humberto Mota de Siqueira: Conceived and designed the experiments; Analyzed and interpreted the data.

Milton Sergio Fernandes de Lima: Conceived and designed the experiments; Analyzed and interpreted the data; Wrote the paper.

### Funding statement

This work was supported by the Coordenação de Aperfeiçoamento de Pessoal de Nível Superior – Brasil (CAPES), and also by the São Paulo Research Foundation (FAPESP) (grants 2019/25229-7 and 2016/11309-0).

### Data availability statement

Data will be made available on request.

### Declaration of interests statement

The authors declare no conflict of interest.

### Additional information

No additional information is available for this paper.
